# Examining the Effects of Ostracism and Overinclusion in the Eating Disorder Binge Spectrum: Evidence From a Controlled Cyberball Task

**DOI:** 10.1002/erv.70007

**Published:** 2025-07-03

**Authors:** Paolo Meneguzzo, Alberto De Mico, Enrico Collantoni, Valentina Meregalli, Elena Tenconi, Angela Favaro

**Affiliations:** ^1^ Department of Neuroscience University of Padova Padova Italy; ^2^ Padova Neuroscience Center University of Padova Padova Italy; ^3^ Department of General Psychology University of Padova Padova Italy

**Keywords:** binge eating disorder, bulimia nervosa, cyberball, early maladaptive schema, eating disorder, ostracism, overinclusion

## Abstract

**Objective:**

Eating disorders (ED), including bulimia nervosa (BN) and binge eating disorder (BED), are characterised by dysfunctional eating behaviours and maladaptive emotional regulation tied to concerns about body image. This study investigates the emotional and cognitive impacts of social exclusion and overinclusion on individuals with ED using the Cyberball task, a computerised simulation of social interaction, to examine the role of early maladaptive schemas (EMS) in these responses.

**Method:**

The sample included 124 women: 34 with BN, 26 with BED, and 64 controls, assigned to either an ostracism or overinclusion condition. Emotional responses were measured using PANAS before and after the Cyberball task, and EMS were assessed via the Young Schema Questionnaire. Mediation models were applied to explore EMS effects on emotional responses.

**Results:**

Participants with ED showed unique emotional patterns compared to controls across both Cyberball conditions. In the overinclusion condition, controls experienced increased positive affect, whereas individuals with ED showed no significant change. During ostracism, ED participants reported reduced negative affect, suggesting complex reactions to social exclusion. EMS were found to mediate emotional responses, particularly in the overinclusion condition.

**Discussion:**

The findings underscore the distinct social‐cognitive and emotional effects of interpersonal interactions on individuals with BN and BED, emphasising the importance of addressing EMS and social cognition in treatment. Future research should focus on refining our understanding of social perception and emotional skills in ED populations, particularly within the bulimic spectrum.

## Summary

- Emotional and cognitive shifts emerged during social interaction tasks.
- Individuals with binge‐spectrum felt less belonging and self‐esteem when overincluded.
- Emotional responses differed between clinical and control groups.
- Negative self‐beliefs explained mood changes after social interactions.
- Targeting self‐beliefs may improve emotional regulation in eating disorders.

## 1 Introduction

Difficulties in emotional regulation and interpersonal functioning are central to eating disorder (ED) psychopathology. Individuals with bulimia nervosa (BN) and binge eating disorder (BED) often struggle to identify their feelings and manage interpersonal relationships (Christensen and Haynos 2020). Social support and connection are critical for mental health and well‐being, while social isolation is a significant risk factor for both physical and mental health problems (Meneguzzo et al. 2020, 2024). In social psychology, ostracism, being excluded and/or ignored within social interactions, causes significant pain and distress, affecting self‐esteem and sense of belonging (Hartgerink et al. 2015; Williams 2007). Individuals with ED appear to be particularly sensitive to social rejection, often misinterpreting ambiguous social signals as rejection (Cardi et al. 2013, 2017). For those with BN and BED, fear of social evaluation and meal‐related anxiety can lead to social withdrawal and dependence on binge eating as a maladaptive coping strategy (Arcelus et al. 2013; Meneguzzo et al. 2023).

Interestingly, social anxiety in EDs may also extend to a fear of positive evaluation. High levels of perfectionism, concerns about mistakes, and others' expectations can amplify this anxiety, contributing to the maintenance of disordered behaviours (Levinson et al. 2019; Levinson and Rodebaugh 2012). Research on emotion processing in EDs shows that individuals often exhibit a blunted response to positive emotions, possibly as a control strategy. This avoidance of emotions, both positive and negative emotions—reflects a limited capacity to process and regulate emotions effectively. For individuals with BN, binge eating can serve as a maladaptive response to both negative and positive emotional states, highlighting difficulties in managing emotional arousal (Fresco et al. 2007; Gilbert and Miles 2000; Selby et al. 2008).

To study the impact of social dynamics on EDs, the Cyberball task offers a robust paradigm. This computerised ball‐tossing game manipulates social inclusion and exclusion by varying how often participants receive the ball (Williams and Jarvis 2006). Exclusion occurs when participants receive the ball infrequently, while overinclusion involves receiving it more often than expected, which can feel awkward (Telesca et al. 2024). This paradigm has been used to evaluate these conditions in both general and clinical populations, including those with autism spectrum disorders, borderline personality disorder, major depression, and social anxiety (Heeren et al. 2017; Sebastian et al. 2009; Seidl et al. 2020). For example, individuals with anorexia nervosa (AN) showed heightened sensitivity to exclusion, overestimating their exclusion compared to controls, but responded similarly to controls under overinclusion (Meneguzzo et al. 2020). In contrast, individuals with bulimia nervosa (BN) and binge eating disorder (BED) exhibit varied emotional and cognitive responses to overinclusion and exclusion, which may be influenced by unique psychopathological features such as heightened impulsivity, greater emotion dysregulation, increased interpersonal sensitivity, and fear of negative evaluation (Duarte et al. 2016; Ivanova et al. 2015; Levinson and Rodebaugh 2012; Meneguzzo et al. 2023).

These social interactions are often filtered through early maladaptive schemas (EMS)o, which represent enduring, negative beliefs about oneself and others developed in response to adverse experiences (Bach et al. 2018; Kaya et al. 2015). EMSs have been implicated in EDs, with evidence suggesting that these schemas mediate the relationship between early trauma and disordered behaviours, such as impulsivity and emotional dysregulation (Estévez et al. 2019; Meneguzzo, Cazzola, et al. 2021; Zhu et al. 2016). For individuals with ED, EMS can distort social interactions and exacerbate emotion regulation difficulties, perpetuating maladaptive coping strategies (Edwards et al. 2021). In this context, it can be hypothesised that EMSs may serve as mediators, explaining the process through which social exclusion or overinclusion impacts emotional responses. Specifically, EMSs may elucidate how these social experiences lead to stronger emotional reactions in people with eating disorders.

This study aimed to investigate the relationship between social dynamics, emotional responses, and cognitive schemas in people with BN and BED. Building on previous work using the Cyberball paradigm in eating disorder populations (Meneguzzo et al. 2023), the present study shifts the focus from behavioural urges and stress perception to the role of early maladaptive schemas (EMSs) in shaping emotional reactions to social interactions.

Specifically, the first objective was to assess the emotional and cognitive impact of ostracism and overinclusion in people with BN and BED compared to healthy controls. The second objective was to explore whether EMSs mediate these emotional responses, and whether this mediation differs by diagnostic group.

Based on previous findings, we hypothesised that individuals with eating disorders would show blunt increases in positive affect during overinclusion and less pronounced increases in negative affect during ostracism, compared to controls. We also expected that participants with BN and BED would report lower feelings of belonging, self‐esteem, and meaningful existence in both social conditions. Finally, we hypothesised that EMSs would mediate the relationship between diagnostic group and emotional response, particularly in the overinclusion condition.

## 2 Methods

### 2.1 Participants

The study involved 124 cisgender women, including 34 diagnosed with BN and 26 with BED, according to DSM‐5 diagnostic criteria. The remaining 64 participants were from the general population and were used as comparison peers (CP). Control participants were recruited using a convenience sampling method, to ensure age comparability with clinical groups. The inclusion criteria for the clinical groups were: (a) female gender, (b) aged 15–35 years, (c) absence of severe psychiatric comorbidities, defined as psychosis or bipolar disorder, (d) no history of neurological trauma or disorders or drug dependence. The inclusion criteria for the control group were: (a) the absence of a lifetime diagnosis of eating disorder, anxiety disorder, or mood disorder, and (b) aged 15–35 years.

A portion of the sample partially overlaps with a previous study (Meneguzzo et al. 2023); however, the present research addresses different hypotheses, focusing on EMS and emotional well‐being rather than food‐related attitudes and the urge to binge. This distinction is now explicitly acknowledged to clarify the novelty of the current report.

All participants or their parents if minors, provided informed consent. The study was approved by the local Ethics Committee and complies with the Declaration of Helsinki.

### 2.2 Procedures

A single‐session randomized study was conducted, involving the random allocation of participants to the ostracism or overinclusion condition. First, participants completed a series of self‐reported questionnaires and specific demographic information was collected. The baseline evaluation included the eating disorder examination questionnaire (EDE‐Q) and the Young schema questionnaire (YSQ‐S3). They then completed the Positive and Negative Affect Schedule (PANAS) evaluation, in which individuals indicate to what extent certain adjectives describe them at that time. They were verbally informed that they would participate in a computer game with two other people in a nearby room. After the task, the participants completed the Need Threat Scale (NTS) and were re‐assessed with PANAS. Finally, all participants were debriefed on the deception and the true purpose of the task, following international guidelines (Wendler and Miller 2004).

### 2.3 Measures

The EDE‐Q (Fairburn and Beglin 1994) is a self‐report questionnaire consisting of 28 items that investigate eating psychopathology and behaviours related to eating disorders that occur 28 days before completion, such as objective and subjective binge eating, vomiting, excessive exercise, laxative and diuretic use. The questionnaire produces a total score and four subscales: restraint, eating concern, weight concern, and shape concern. In the current study, Cronbach's alphas were between 0.868 and 0.937.

The YSQ Short Form third Edition (Young et al. 2005) is a self‐report questionnaire consisting of 90 items that assess maladaptive schemas in four domains: disconnection and rejection; impaired autonomy and performance; excessive responsibility and standards; and compromised limits. A Likert scale of 1–6 is used, with higher scores indicating a greater presence of early maladaptive schemas (EMS). In the current study, the Cronbach alphas for the domains were between 0.864 and 0.925.

PANAS (Watson et al. 1988) is a self‐assessment measure consisting of 20 items aimed at assessing positive and negative affective states. Using a Likert scale, the individual assesses to what extent each adjective describes their feelings at that specific moment. Items are grouped into two different scales: positive PANAS, which indicates how active, enthusiastic, and determined the person feels, and negative PANAS, which reflects a dimension of subjective distress experienced by the individual, for example, in terms of fear, shame, guilt. Although PANAS was originally developed to assess positive and negative affect as independent dimensions, we also computed a total score as a composite index of overall affective state, with higher scores indicating a more positive emotional balance. This approach has been used in prior research to summarise global mood changes across conditions. In the current study, Cronbach's alphas for the domains were 0.867 and 0.912.

NTS (Gerber et al. 2017) is a scale consisting of 20 items used after the Cyberball task, as it reflects the distress following ostracism. Responses are reported on a five‐point Likert scale, where higher scores indicate greater feelings of threat and discomfort, divided into four dimensions: belongingness, self‐esteem, meaningful existence, and manipulation check. The Manipulation Check subscale assesses the participant's awareness and perception of social inclusion or exclusion manipulation during the Cyberball task. This ensures that the observed emotional responses are based on the participant's experience of the task conditions. In addition to the four subscales, we computed a focused ostracism score based on the first five items of the NTS, which specifically assess perceived exclusion (e.g., ‘I felt like an outsider,’ ‘I was ignored’). These items have been used in prior research as a direct measure of ostracism perception (Meneguzzo et al. 2020). Higher scores indicate greater feelings of being ostracised. In the current study, the Cronbach alphas for the domains were between 0.725 and 0.788.

### 2.4 The Cyberball Task

The Cyberball Task is a computerised ball‐tossing game that evaluates the psychological effects of ostracism and overinclusion within a virtual social situation (Williams and Jarvis 2006). Participants engage in a preset exposure, varying depending on the condition to which they are assigned, but are told that they will play an online ball tossing game with two unknown individuals. In this task, the other two participants are represented by female names, which helps maintain the social context and ensures the interaction appears to be with other women. The decision to represent the other participants in the Cyberball task with female names was made for simplicity, as the study focused exclusively on cisgender women and is an exploratory approach to the field. The task lasted about 5 min and involved 30 throws. In the social exclusion condition, participants received the ball 4 times (13% of total throws). In the overinclusion condition, 15 tosses were decided by the participant, who chose to pass the ball to one of the other two participants. The remaining 15 tosses were predetermined by the task: 14 were directed to the participant being tested and only 1 was assigned to the other virtual participant. The percentage of ball reception was decided based on previous literature on Cyberball in various psychiatric populations (De Waal‐Andrews and Van Beest 2020; Niedeggen et al. 2014; Telesca et al. 2024).

### 2.5 Statistical Analyses

Due to the nonnormal distribution of most variables, nonparametric Wilcoxon‐Mann‐Whitney tests were used to evaluate demographic and psychological variables. For repeated measures (pre‐ and post‐Cyberball PANAS scores), a Friedman test was used. Separate mediation analyzes were performed for each condition to assess whether the maladaptive schemas predicted emotional changes, evaluating subtracting pre‐to post‐Cyberball PANAS scores (Δ PANAS). To evaluate the interaction between the conditions of exclusion and overinclusion and various psychological features assessed, several ANOVA analyzes were conducted, allowing for a comprehensive examination of how these factors relate to each other. Mediation models were evaluated using the SPSS PROCESS macro, specifically Model 4. The bootstrapping sampling distributions for indirect effects were set at 5,000, and the bias‐corrected confidence intervals were set at 95%. The effect sizes were calculated using eta‐squared (*η* ^2^) and *k* ^2^ for the mediation models. The significance level was established at *p* < 0.05 for all analyzes. All analyzes were performed using IBM SPSS Statistics 25.0 (SPSS, Chicago, IL, United States).

## 3 Results

### 3.1 Characteristics of the Sample

Table 1 includes the descriptive indices of the variables in the study. Differences between clinical and comparison participants were found in all psychological evaluations between the main groups, while no differences emerged between subgroups compared by conditions.

**TABLE 1:** Description of the sample.

	CP	ED	*Z*	*p*	CP	ED
	*n* = 64	*n* = 60		*η* ^2^	E vs. O	E vs. O
Age, years	24.31	25.60	−1.127	0.260	0.139	*p* = 0.417
	(3.20)	(8.52)		0.01		
BMI, kg/m2	22.95	24.29	−0.710	0.478	*p* = 0.891	*p* = 0.225
	(3.56)	(5.47)		< 0.01		
Education, years	15.28	14.45	−1.840	0.070	*p* = 0.101	*p* = 0.372
	(2.27)	(2.72)		0.03		
EDEQ						
Restraint	1.06	2.49	−4.587	< 0.001	*p* = 0.475	*p* = 0.063
	(1.11)	(1.80)		0.17		
Eating concerns	0.47	3.15	−8.374	< 0.001	*p* = 0.162	*p* = 0.599
	(0.78)	(1.51)		0.57		
Shape concerns	1.65	4.35	−7.612	< 0.001	*p* = 0.698	*p* = 0.866
	(1.39)	(1.41)		0.47		
Weight concerns	1.19	3.59	−7.074	< 0.001	*p* = 0.431	*P* = 0.572
	(1.13)	(1.63)		0.41		
Global score	1.09	3.40	−7.847	< 0.001	*p* = 0.445	*p* = 0.617
	(1.10)	(1.59)		0.50		
YSQ						
DR	0.35	1.17	−5.358	< 0.001	*p* = 0.532	*p* = 0.681
	(0.46)	(0.93)		0.23		
IAP	0.39	1.38	−6.142	< 0.001	*p* = 0.576	*p* = 0.728
	(0.52)	(1.10)		0.31		
ERS	0.40	1.04	−5.131	< 0.001	*p* = 0.275	*p* = 0.860
	(0.42)	(0.72)		0.21		
IL	0.56	1.42	−5.621	< 0.001	*p* = 0.464	*p* = 0.561
	(0.56)	(0.88)		0.26		
EMS total score	0.42	1.25	−6.973	< 0.001	*p* = 0.387	*p* = 0.908
	(0.42)	(0.83)		0.40		
PANAS						
Positive	30.87	26.12	−4.247	< 0.001	*p* = 0.097	*p* = 0.200
	(5.11)	(6.31)		0.15		
Negative	12.30	15.92	−2.583	0.010	*p* = 0.590	*p* = 0.932
	(4.36)	(7.46)		0.05		
PANAS total	43.17	42.03	−0.140	0.888	*p* = 0.302	*p* = 0.688
	(6.26)	(7.02)		< 0.01		

*Note:* For *η* ^2^: 0.01 indicates a small effect, 0.06 indicates a medium effect, and 0.14 indicates a large effect. E versus. O: exclusion versus. overinclusion condition (*p*‐values are reported in the coloums).

Abbreviations: CP, comparison peer; DR, disconnection and rejection domain; ED, eating disorder; ERS, excessive responsibility and standard domain; IAP, impaired autonomy and performance domain; IL, impaired limits domain.

The final sample included 124 cisgender women, with 62 participants assigned for the ED group (30 to the exclusion condition and 32 for the overinclusion condition) and 62 to the CP group (32 for the exclusion condition and 30 for the overinclusion condition).

### 3.2 The Cyberball Effects

Different effects of the Cyberball task were found using the specific NTS scale, with the ED groups reporting lower scores in both exclusion and over‐inclusion compared to controls. See Table 2.

**TABLE 2:** Need‐threat scale after each cyberball condition.

	Exclusion condition			Overinclusion condition			CP vs. CP	ED vs. ED
	CP	ED	*Z*	CP	ED	*Z*	*Z*	*Z*
	*n* = 32	*n* = 30	*p*	*n* = 32	*n* = 30	*p*	*p*	*p*
% Passages	21.1	16.7	−1.999	55.9	62.5	−2.278	−6.853	−6.660
	(7.2)	(8.7)	0.049	(12.0)	13.2)	0.023	< 0.001	< 0.001
Belonging	12.91	12.87	−0.297	21.47	19.70	−2.432	−6.540	−5.695
	(3.07)	(3.75)	0.766	(2.90)	(3.51)	0.015	< 0.001	< 0.001
Self‐esteem	14.00	12.00	−2.569	18.16	15.57	−2.187	−4.235	−2.791
	(3.35)	(3.91)	0.010	(3.47)	(4.65)	0.029	< 0.001	0.005
Meaningful existence	14.53	12.90	−1.885	20.50	18.80	−2.584	−6.232	−5.495
	(3.15)	(3.55)	0.059	(2.57)	(2.83)	0.010	< 0.001	< 0.001
Control	9.59	8.53	−1.567	16.38	13.30	−3.587	−6.042	−4.592
	(2.46)	(2.97)	0.117	(3.19)	3.56	< 0.001	< 0.001	< 0.001
Manipulation check	6.28	6.67	−0.501	2.09	2.13	−0.606	−6.826	−6.532
	(2.37)	(2.32)	0.616	(0.53)	(0.51)	0.544	< 0.001	< 0.001

Abbreviations: CP, comparison peer; ED, eating disorder.

In the CP group, all NTS subscales showed higher scores in the overinclusion condition compared to the ostracising condition, a pattern mirrored in the ED group, as shown in Table 2.

The interaction between condition (exclusion vs. overinclusion) and group (ED vs. CP) was significant on all NTS subscales (see Table 3).

**TABLE 3:** Interaction effects: Condition × group on need‐threat scale subscales.

Need‐threat scale subscale	*F*	*p*
% passages	152.97	< 0.001
Belonging	57.69	< 0.001
Self‐esteem	14.06	< 0.001
Meaningful existence	42.47	< 0.001
Control	42.91	< 0.001
Manipulation check	67.97	< 0.001

*Note:* Higher scores indicate greater threat to basic psychological needs (e.g., belonging, self‐esteem). Significant Condition × Group interactions suggest differential responses to exclusion and overinclusion between ED and CP groups.

Abbreviations: CP, comparison peers; ED, eating disorder group.

### 3.3 Emotional Changes During the Cyberball Task

In the ostracised condition, the CP group exhibited a significant decrease in positive affect and an increase in negative affect, with no change in the total score. In the ED group, the positive affect decreased significantly, the negative affect remained stable and the total score decreased.

**FIGURE 1:**
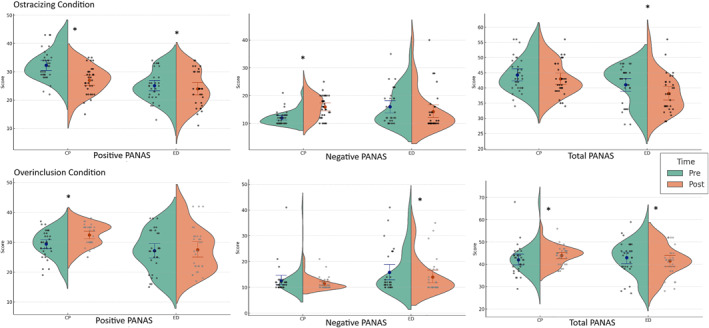
Violin plots of PANAS scores (Positive, Negative, and Total) across conditions (Ostracising and Overinclusion), groups (Comparison Peers [CP] and Eating Disorders [ED]), and timepoints (Pre vs. Post). Each violin represents the distribution of scores, with individual dots for participants, mean scores (dots), and 95% confidence intervals (bars). * indicate statistically significant differences between Pre and Post scores within each group (*p* < 0.05).

In the overinclusion condition, the CP group showed a significant increase in positive affect and total score, while negative affect remained stable. In the ED group, negative affect decreased significantly, with a corresponding decrease in total score, while positive affect remained stable (see Table 4 for details). Figure 1 illustrates these emotional changes between conditions and groups.

**TABLE 4:** Emotional changes from cyberball task.

Group	PANAS	Pre	Post	*Z*	*p*	Change
Ostracised condition						
CP	Positive	30.87	29.73	−4.716	< 0.001	↓
		(5.11)	(5.13)			
CP	Negative	12.30	13.73	−4.163	< 0.001	↑
		(4.36)	(4.02)			
CP	Total score	44.28	42.97	−1.363	0.173	NS
		(5.60)	(5.41)			
ED	Positive	26.12	25.74	−2.826	0.005	↓
		(6.31)	(7.29)			
ED	Negative	15.92	14.45	−1.211	0.226	NS
		(7.46)	(7.31)			
ED	Total score	41.07	38.72	−2.979	0.003	↓
		(6.25)	(6.72)			
Overinclusion condition						
CP	Positive	29.47	32.47	−3.491	< 0.001	↑
		(4.51)	(3.69)			
CP	Negative	12.59	11.50	−1.392	0.164	NS
		(5.68)	(2.50)			
CP	Total score	42.06	43.97	−2.874	0.004	↑
		(6.76)	(4.28)			
ED	Positive	27.17	27.18	−1.177	0.239	NS
		(7.01)	(7.43)			
ED	Negative	15.83	14.32	−3.229	0.001	↓
		(8.40)	(6.99)			
ED	Total score	43.00	41.50	−2.667	0.008	↓
		(7.70)	(7.01)			

*Note:* PANAS scores presented as means (SD). Arrows indicate direction of change from pre‐to post‐task.

Abbreviations: CP, comparison peers; ED, eating disorder group. ↑, significant increase; ↓, significant decrease; NS, non‐significant.

A time‐by‐condition interaction effect was observed for the CP group on both positive and negative PANAS subscales, but not for the ED group. Additionally, the three‐way interaction of time, condition, and group was significant for both PANAS subscales.

### 3.4 Predicting the Effects of Maladaptive Schemas

**FIGURE 2:**
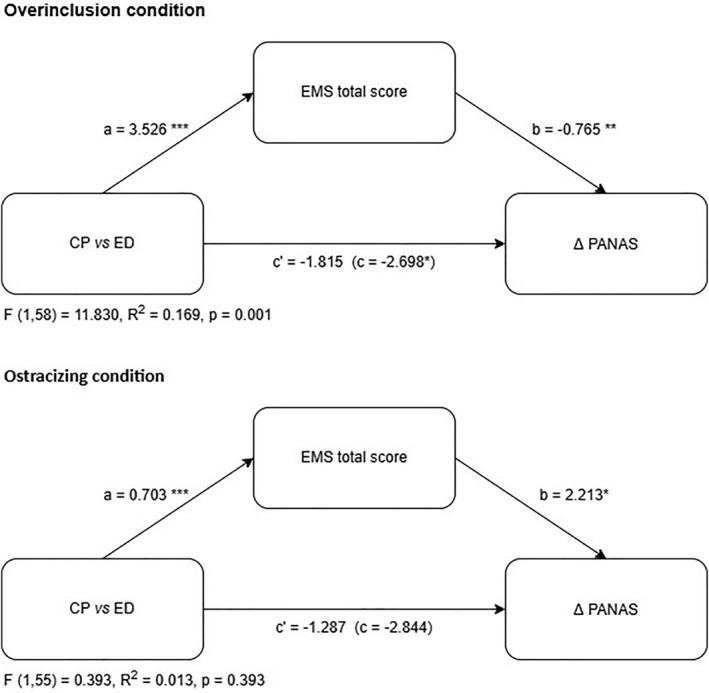
The total effect of the group (ED = 1 vs. CP = 0) on the emotional change following the inclusion of the Cyberball condition and the mediation analysis of this effect through early maladaptive schemas. Note. Standardized coefficients. Path c’ is the total effect, path c represents the direct effect of the group, and paths a and b are the indirect effects. Δ: changes in PANAS scores (post‐Cyberball—pre‐Cyberball). *p < 0.05, **p < 0.01, ***p < 0.001.

The influence of maladaptive schema domains on ΔPANAS varied by domain. The Disconnection and Rejection, Excessive Responsibility and Impaired Limits domains significantly predicted changes between conditions, with interaction effects present for specific domains (Table 5). Mediation analysis revealed that in the overinclusion condition, total EMS scores mediated the relationship between group (ED vs. CP) and emotional changes. On the contrary, no mediation effects were found in the ostracising condition. A graphical representation of the mediation is shown in Figure 2.

**TABLE 5:** Predicting effects of maladaptive schemas on ΔPANAS.

Domain	*F*	*p*	Condition × group interaction	*p*
Disconnection and rejection	32.035	< 0.001	6.083	0.016
Impaired autonomy and performance	25.220	0.001	1.042	0.379
Excessive responsibility	16.090	0.014	6.390	0.001
Impaired limits	22.934	0.002	1.588	0.210

*Note:* EMS domains refer to early maladaptive schema domains from the Young Schema Questionnaire. ΔPANAS = change in affect score (post − pre). Significant Condition × Group interactions suggest schema‐related differences in emotional reactivity between ED and CP groups.

## 4 Discussion

This study aimed to investigate changes in emotional and cognitive aspects related to eating disorders (specifically, in the binge spectrum) after experiencing social inclusion or exclusion scenarios. This was achieved through the Cyberball task, a computerised ball‐throwing game that alternately presented one of two conditions to the participants. This is the first study conducted in patients with BN and BED that, in addition to examining the effects of social exclusion and overinclusion, considered the possible influence of EMS in mediating the impact of the experience on mood aspects. The objective was to identify any similarities or differences compared to a previous study conducted in individuals with AN and with different conditions (Meneguzzo et al. 2020; Telesca et al. 2024).

First, the results emphasise the significant impact of the Cyberball task. Substantial changes in levels of belonging, self‐esteem, meaningful existence, control, and manipulation checks were observed before and after the task, depending on the condition assigned to the participants. This effect followed the same pattern in both the control and clinical groups for overinclusion and ostracism. However, it is important to note the significant psychological impact of overinclusion: participants in the binge spectrum reported worse scores on belonging, self‐esteem, meaningful existence, and control than healthy controls, except for the manipulation check. This finding is in contrast to other psychiatric conditions, in which improved psychological functioning has been reported following social inclusion (Telesca et al. 2024). The literature is still preliminary, and our report is the first in this field, necessitating replication. Our findings might suggest that inclusion and overinclusion may not always result in positive experiences, particularly in the context of eating disorders, due to their specific impact on psychopathology (Ivanova et al. 2015). These results also underscore the importance of social interactions in eating psychopathology, with the interpersonal model suggesting that social feedback can exacerbate maladaptive schemas related to self‐worth and body image (Christensen and Haynos 2020; Goddard et al. 2 011). Such interactions may trigger emotions like shame, anger, and anxiety, which reinforce disordered eating behaviours and maintain the disorder (Duarte et al. 2014, 2016; Grabhorn et al. 2006; Todisco et al. 2023).

An intriguing finding of this study was the apparent divergence between the PANAS and NTS outcomes in the overinclusion condition. While individuals with EDs showed a significant reduction in negative affect on the PANAS, they simultaneously reported lower scores in perceived belonging, self‐esteem, meaningful existence, and control on the NTS compared to controls. This suggests that participants may have experienced a reduction in immediate negative emotional arousal, yet cognitively appraised the interaction as interpersonally threatening or uncomfortable. One possible explanation lies in emotional avoidance or suppression, common in ED populations, where affective responses are downregulated even as underlying cognitive schemas remain activated (Leppanen et al. 2022; Prefit et al. 2019). Another possibility is that alexithymic traits or low interoceptive awareness—prevalent in individuals with BN and BED—limit the conscious recognition of negative emotional states, resulting in an emotional ‘numbing’ that coexists with negative social appraisals (Meneguzzo, Garolla, et al. 2022; Meneguzzo, Mancini, et al. 2022). Lastly, early maladaptive schemas, particularly those related to defectiveness and mistrust, can influence the interpretation of inclusion as suspicious or undeserved, diminishing the perceived value of the interaction despite reduced emotional distress (Aloi et al. 2015, 2017; Todisco et al. 2023). Future studies should explore this dissociation further using multimodal assessments, including qualitative or physiological measures.

Moreover, we evaluate the effects of being included or ostracised on emotions. In the overinclusion condition, although there is an increase in positive emotions for the control group, this is not observed in individuals with BN and BED. This result partly overlaps with observations in patients with AN (Meneguzzo et al. 2020) and may indicate difficulties in processing social inclusion within these populations. The lack of a progressive increase in positive emotions in the binge spectrum as inclusion increases could be related to the presence of a judgemental atmosphere (Brown and Levinson 2022; Grabhorn et al. 2006). Social comparison and judgement are believed to contribute to the psychopathology of eating disorders, influencing overall functioning (Patel et al. 2016). Furthermore, interpersonal vulnerability and emotional difficulties in the ED are crucial elements to consider during treatment across the spectrum of the ED (Meneguzzo, Garolla, et al. 2022; Treasure and Todd 2016), which can also interact with body shame and judgements (Duarte et al. 2014). These findings can also be contextualised within the framework of mental health stigma, which plays a central role in shaping interpersonal experiences for individuals with EDs (O’Connor et al. 2021). Repeated exposure to stigmatising attitudes—whether through overt social exclusion or more subtle forms of marginalisation—may foster internalized stigma, in which individuals adopt negative societal views about their disorder (Brelet et al. 2021). This self‐stigmatisation has been associated with increased shame, lower self‐esteem, and a greater fear of social rejection (Griffiths et al. 2015; Puhl and Suh 2015). As a result, positive social interactions such as overinclusion may be interpreted not as affirming, but as potentially threatening or inauthentic, causing discomfort rather than reassurance. This could partly explain the blunted affective responses observed in the clinical group, particularly in contexts where positive social signals would normally elicit increased positive affect. In this light, emotional detachment can serve as a defencive mechanism to avoid further social injury, especially in individuals whose early maladaptive schemas reinforce beliefs of defectiveness, unlovability, or social exclusion (Aloi et al. 2025; Monteleone, Cardi, et al. 2018). Addressing both stigma and schema‐based cognitive vulnerabilities may therefore be crucial for improving social connectedness and emotional regulation in ED treatment.

Looking at the ostracism condition, a decrease in positive emotions was observed in the clinical group, compared to the control group, which reported an increase in negative emotions. This finding aligns with research conducted in post‐bariatric patients (Meneguzzo, Tenconi, et al. 2021) and supports the idea of a connection between social isolation and eating psychopathology (Southward et al. 2014). Even minimal participation in a social activity, such as the Cyberball task, may be enough to produce positive psychological effects for non‐clinical individuals. The tendency to feel socially excluded has been linked to binging (Mason et al. 2016) and harms psychological well‐being (Steiger et al. 1999).

The different emotional trends in the two groups, depending on whether they experienced ostracism or overinclusion, are evident. However, in our study, we also observed that this is not a direct relationship. The role of EMS explains this difference, but only in the overinclusion condition. EMS played a mediating role in mood differences in the overinclusion condition, highlighting its potential role in the emotional responses of individuals with eating disorders, as proposed by preliminary evidence (Maher et al. 2021). EMSs are considered possible vulnerabilities for the development of psychological vulnerabilities and for specific disorders, emerging as potential targets for clinical interventions (Bär et al. 2023). In our experimental condition, a complete mediation was found, indicating that without considering EMS patterns, there is no evidence of a clinically significant outcome. This finding points to a promising area for clinical intervention, where addressing the interaction between eating disorders and social experiences could improve emotional regulation. Cognitive models of ED have been proposed for treatments (Legenbauer et al. 2018; Pauwels et al. 2018), but the research is still preliminary. Our findings might contribute to the development of treatment approaches that integrate social cognition and maladaptive schemas, with the aim of effective changes in emotional vulnerabilities (Gerges et al. 2022; Joshua et al. 2024). Although we used the total EMS score in the mediation model to reduce complexity and intercorrelation between domains, the interaction found with the Excessive Responsibility domain suggests that certain schemas may have a stronger role in shaping emotional responses. Future research should explore whether specific schema profiles—such as excessive responsibility, defectiveness, or social isolation—differentially mediate responses to social inclusion or exclusion.

The observed blunted or paradoxical emotional responses to overinclusion in individuals with BN and BED may be partially explained by the interplay between early maladaptive schemas and social anxiety. In a recent meta‐analytic review of Cyberball studies was found that individuals with high social anxiety exhibited greater increases in positive affect during overinclusion compared to those with low social anxiety (Hay et al. 2023). This suggests that socially anxious individuals may be more sensitive to positive social cues, possibly due to their heightened concern about social evaluation. Given that certain EMSs, such as those related to social isolation or defectiveness, are associated with social anxiety, it is plausible that these schemas contribute to the atypical emotional responses observed in our study. Future research should explore the mediating role of specific EMS domains in the relationship between social anxiety and emotional reactions to social inclusion and exclusion.

These findings can also be considered in relation to previous research using the Cyberball paradigm with individuals diagnosed with anorexia nervosa (Meneguzzo et al. 2020; Telesca et al. 2024). In those studies, individuals with AN showed heightened emotional sensitivity to social exclusion, often overestimating the degree to which they were being excluded, but reacted similarly to healthy controls in overinclusion conditions. In contrast, participants with BN and BED in the present study displayed more complex or attenuated responses, particularly to overinclusion, where they did not experience the expected increase in positive affect observed in the control group. This might suggest that while both AN and binge‐spectrum EDs are characterised by interpersonal sensitivity, the nature and expression of this sensitivity may differ: individuals with AN may be hypervigilant to exclusion cues, whereas those with BN and BED may experience ambivalence or discomfort even in situations of social acceptance. These differences could be linked to disorder‐specific features such as impulsivity, shame, and difficulties in processing positive social feedback, and they underscore the importance of tailoring interpersonal models and therapeutic approaches to the specific relational vulnerabilities of each ED subtype (Christensen and Haynos 2020; Murphy et al. 2012; Treasure and Todd 2016).

It is worth emphasising the strengths of this study, as it is the first to explore the issue of emotional response to inclusion. We align with recent evidence from systematic revision of the literature (Telesca et al. 2024) on the importance of investigating even seemingly positive conditions. The methodology employed well‐validated instruments, enhancing the study's rigour and reliability. Although these strengths, several limitations must be considered. We adopted a transdiagnostic approach to the binge spectrum, acknowledging that specific differences may arise between subgroups with and without compensatory behaviours, as well as between different ages. A limitation of this study is the sample size, as a larger and more diverse sample, including individuals of different genders, would be desirable to enhance the generalisability of the findings. Additionally, the study focused solely on cisgender women, and the Cyberball task was designed to simulate interactions within a female‐oriented context, which may limit the applicability of the results to other gender groups. Finally, the lack of correction for multiple comparisons may increase the risk of Type I error; however, given the exploratory design of the study, these analyses were intended to generate hypotheses for future confirmatory research. Future studies could benefit from integrating open‐ended questions to explore participants' subjective interpretations of the inclusion conditions. In particular, understanding why overinclusion feels uncomfortable may help clarify how interpersonal expectations and internal schemas contribute to atypical affective responses in ED populations. Moreover, they might employ different strategies to assess emotional and cognitive responses, including neurophysiological measures.

## 5 Conclusions

This study examined the effects of ostracism and overinclusion in the binge eating spectrum of eating disorders, focussing on the specific effects of EMS on emotional responses. The results underscored the different impacts that exclusion and overinclusion could have on individuals with eating disorders, supporting existing interpersonal models for ED and highlighting the need for more specific evaluations of positive or negative perceptions of social evaluations. The mediation effects of EMS suggest the possible role of cognitive schemas in social interactions between individuals with ED, indicating a potential area for therapeutic research. Future research should focus on refining therapy models to address interpersonal and emotional difficulties, as well as cognitive mechanisms, to improve the efficacy of treatment for those with eating disorders.

## Author Contributions

Conceptualization: P.M., E.T. and A.F., methodology: P.M., software: P.M., validation: P.M., V.M. and E.C., formal analysis: P.M., investigation: P.M., resources: A.F., data curation: P.M., writing – original draft preparation: P.M., A.D.M., V.M. and E.C., writing – review and editing: E.T. and A.F., visualization: P.M., supervision: E.T. and A.F., project administration: A.F., funding acquisition: A.F. All authors contributed to and approved the final manuscript.

## Ethics Statement

All individuals included in this case series provided informed consent. The study protocol received approval from the local Ethics Committee and complies with the ethical principles outlined in the Declaration of Helsinki and its subsequent amendments.

## Conflicts of Interest

The authors declare no conflicts of interest.

## Data Availability

Data will be made available on request.
